# Impact of Drying Methods on β-Glucan Retention and Lipid Stability in Oyster Mushroom (*Pleurotus ostreatus*) Enriched Carp (*Cyprinus carpio*, L.) Fish Burgers

**DOI:** 10.3390/molecules30234649

**Published:** 2025-12-03

**Authors:** Grzegorz Tokarczyk, Katarzyna Felisiak, Iwona Adamska, Sylwia Przybylska, Agnieszka Hrebień-Filisińska, Patrycja Biernacka, Grzegorz Bienkiewicz, Małgorzata Tabaszewska, Emilia Bernaś, Eire López Arroyos

**Affiliations:** 1 Department of Fish, Plant and Gastronomy Technology, Faculty of Food Sciences and Fisheries, West Pomeranian University of Technology in Szczecin, 70-310 Szczecin, Poland; grzegorz.tokarczyk@zut.edu.pl (G.T.); katarzyna.felisiak@zut.edu.pl (K.F.); iwona.adamska@zut.edu.pl (I.A.); sylwia.przybylska@zut.edu.pl (S.P.); agnieszka.filisinska@zut.edu.pl (A.H.-F.);; 2 Department of Commodity Science, Quality Assessment, Process Engineering and Human Nutrition, Faculty of Food Sciences and Fisheries, West Pomeranian University of Technology in Szczecin, 70-310 Szczecin, Poland; grzegorz.bienkiewicz@zut.edu.pl; 3 Department of Plant Product Technology and Nutrition Hygiene, Faculty of Food Technology, University of Agriculture in Cracow, Balicka Str. 122, 30-149 Cracow, Poland; malgorzata.tabaszewska@urk.edu.pl (M.T.); emilia.bernas@urk.edu.pl (E.B.); 4 Department of Human Nutrition and Metabolomics, Pomeranian Medical University in Szczecin, Broniewskiego Street 24, 71-460 Szczecin, Poland; 5 Department of Food Science and Technology, University of Zaragoza, C. de Pedro Cerbuna Street 12, 50009 Zaragoza, Spain

**Keywords:** fish burger, common carp, lipid oxidation, β-glucans, oyster mushroom

## Abstract

Background: The incorporation of edible mushrooms into fish-based products offers a promising approach to enhance nutritional quality and oxidative stability. Oyster mushrooms (*Pleurotus ostreatus*) are valued for their β-glucans and bioactive compounds. This study aimed to evaluate the effects of hot-air dried and freeze-dried oyster mushrooms, added at different levels, on the nutritional composition, lipid quality, and oxidative stability of carp burgers. Methods: Carp burgers were prepared with 0.5–2.0% (*w*/*w*) of hot-air dried or freeze-dried oyster mushrooms, rehydrated at a standardized ratio of 5:1. Nutritional composition, β-glucan content, fatty acid profile, and lipid oxidation were determined. Oxidative stability was assessed by peroxide, p-anisidine, and total oxidation, while nutritional quality was evaluated using lipid indices, including polyunsaturated-to-saturated fatty acid ratio (PUFA/SFA), and atherogenicity index. Results: Freeze-dried mushrooms preserved higher β-glucan content (5.80 g/100 g at 2% inclusion) than hot-air dried samples (2.21 g/100 g). Their addition lowered fat by 19.6% and enhanced oxidative stability, with peroxide and anisidine values reduced by 23% and 35%, respectively. Lipid nutritional indices improved, as the PUFA/SFA ratio increased by 15% and the atherogenicity index remained below 0.36 across all treatments. At 2.0% inclusion, freeze-dried mushrooms maximized β-glucan retention (96.9%) and reduced TOTOX by 22.2%. The optimal range for balanced oxidative protection was 1.5–2.0%. Conclusions: Incorporating freeze-dried oyster mushrooms at 1.5–2.0% with standardized rehydration improves the nutritional profile, fatty acid composition, and oxidative stability of carp burgers. These results provide practical parameters for developing functional fish products with enhanced health value and extended shelf-life.

## 1. Introduction

The growing demand for healthier and more sustainable food products has contributed to increased investment in innovation in the development of functional foods, particularly in the meat and seafood sector. Fish burgers represent a promising alternative to traditional meat-based products due to their favorable nutritional profile, including high-quality protein and beneficial omega-3 fatty acids [1]. Although commercial formulations most commonly utilize marine species such as salmon, tuna, and cod, freshwater species—especially carp (*Cyprinus carpio* L.)—offer distinct advantages that justify their selection for functional product development. Carp is one of the most widely farmed freshwater species worldwide and plays a central role in European and Asian aquaculture due to its high yield, adaptability, and environmentally efficient production [2]. Its lipid profile, rich in polyunsaturated fatty acids, and its high protein content make it a nutritionally valuable raw material for processed fish products [3]. Moreover, recent research has demonstrated that carp burgers can achieve favorable sensory acceptance when formulated appropriately [4]. However, like other fish-based products, they are susceptible to lipid oxidation, compromising quality, shelf life, and nutritional value [5]. Food technology has increasingly focused on natural additives with antioxidant and functional properties, such as edible mushrooms, to meet this challenge.

Oyster mushrooms (*Pleurotus ostreatus*) are particularly valuable for food enrichment due to their rich composition of bioactive compounds. They are an excellent source of dietary fiber (13–24%), minerals (8–13%), and, most notably, β-glucans, which can constitute up to 40% of their dry weight [6,7]. β-glucans from mushrooms are known for their health-promoting effects, including cholesterol reduction, immunomodulatory activity, and antioxidant capacity [8]. Additionally, oyster mushrooms contain phenolic compounds and ergothioneine, a potent antioxidant, that contributes to their ability to inhibit lipid oxidation [9]. These properties make them an ideal functional ingredient for improving the nutritional quality and oxidative stability of processed foods.

The method of mushroom preservation plays a crucial role in retaining these bioactive compounds. Hot-air drying, while widely used due to its cost-effectiveness, can lead to significant losses of heat-sensitive nutrients, including β-glucans and antioxidants, due to prolonged exposure to high temperatures [10]. In contrast, freeze-drying (lyophilization) minimizes thermal degradation, better preserving the structural integrity and functionality of these compounds [11]. Recent studies have demonstrated that freeze-dried oyster mushroom powder retains significantly higher levels of β-glucans (20–30% more) compared to hot-air-dried samples [12]. This difference is critical when considering the potential health benefits and technological performance of mushroom-enriched food products.

The application of mushrooms in meat and fish products has been explored in previous research, though with varying focuses. For instance, Patinho et al. (2019) demonstrated that incorporating *Agaricus bisporus* mushroom powder into beef burgers reduced fat content while enhancing antioxidant capacity [13]. Similarly, Cerón-Guevara et al. (2020) reported that oyster mushroom flour in sausages not only improved dietary fiber content but also reduced lipid oxidation during storage [14].

However, despite these advances, a lack of systematic studies remains in comparing the effects of different drying methods on the nutritional and oxidative stability of mushroom-fortified fish products, particularly burgers. This knowledge gap highlights the need for research that evaluates how processing techniques affect the functional properties of mushroom ingredients in fish-based formulations.

The mechanisms by which mushrooms enhance lipid stability in food products are multifaceted. First, the antioxidant compounds present in mushrooms, such as phenolics and ergothioneine, act as free radical scavengers, thereby slowing the formation of primary and secondary lipid oxidation products [15]. Second, β-glucans can chelate pro-oxidant metals like iron, which are known to catalyze lipid peroxidation [16]. Third, the incorporation of mushrooms can modify the fatty acid profile of the final product, often increasing the ratio of polyunsaturated to saturated fatty acids (PUFA/SFA), which has been associated with improved oxidative stability and nutritional quality [17]. These combined effects make mushrooms a promising natural alternative to synthetic antioxidants in processed foods.

Given the increasing consumer demand for minimally processed foods formulated without synthetic additives, the development of fish burgers enriched with oyster mushrooms presents a significant opportunity. However, to maximize the benefits of this functional ingredient, it is essential to optimize the processing conditions, particularly the drying method, to preserve its bioactive compounds. This study aims to investigate the impact of hot-air drying and freeze-drying on the retention of β-glucans and other functional components in oyster mushrooms and their subsequent effects on the nutritional quality and lipid stability of carp fish burgers. By comparing these two drying techniques, we seek to provide evidence-based recommendations for the production of healthier, more stable fish products with enhanced functional properties.

The findings of this research will contribute to the growing body of knowledge on the use of mushrooms as functional food ingredients, particularly in fish applications. Additionally, the study will offer practical insights for food manufacturers looking to develop products that meet consumer demands for both—health and convenience. By addressing the critical factors influencing the stability and nutritional value of mushroom-enriched fish burgers, this work supports the broader goal of creating sustainable, nutrient-rich food options for modern diets.

## 2. Results and Discussion

### 2.1. Raw Materials

#### 2.1.1. Basic Characteristics

The basic composition of the raw materials is presented in Table 1. The results clearly show that the drying method affects the proximate composition of oyster mushrooms, with freeze-drying producing markedly lower water content and preservation of protein compared with hot-air drying. The lipid content is low and comparable between the drying methods. The observed trends are in agreement with previous studies on the effects of drying on food composition, further reinforcing the nutritional value of dried oyster mushrooms as an alternative protein source [18]. The observed differences between hot air-dried and freeze-dried mushrooms indicate the higher efficiency of freeze-drying in moisture removal, as reported by Shams et al. (2022) [19].

#### 2.1.2. Determination of Total Glucans Content

In this study, oyster mushrooms are the primary source of β-glucans incorporated into the burger formulations [20]. In the case of oyster mushrooms, they can occur at a level of 40.34 ± 3.23 g/100g of dry weight (DW) [6]. The total glucans content in hot-dried oyster mushrooms is 28.00 ± 0.91 g/100g DW (β fraction 27.86 ± 0.90 and α fraction 0.14 ± 0.03), and in freeze-dried oyster mushrooms 34.35 ± 0.23 g/100g DW (β fraction 34.07 ± 0.25 and α fraction 0.28 ± 0.30). Freeze-drying has been reported to be a safer technique for maintaining glucan stability; however, other thermal processes may adversely affect their final content. High frying temperature and deep oil penetration into fish burgers can cause a significant reduction in β-glucans content [15]. Nevertheless, β-glucans (most often yeast) are popularly added to products such as breads, cookies, and meat products, where high-temperature processing is used, because of the desired physicochemical effects obtained [21].

#### 2.1.3. Determination of Lipid Quality Parameters

The lipid oxidation parameters of the raw materials revealed substantial variation in oxidative stability between carp meat and mushroom samples (Table 2). Freeze-dried oyster mushrooms exhibited markedly lower peroxide values (1.1 ± 0.2 meqO_2_/kg fat) compared to both hot-air dried mushrooms (2.0 ± 0.0 meqO_2_/kg fat) and carp meat (5.1 ± 0.1 meqO_2_/kg fat). The remaining lipid values showed a similar trend. The thermal degradation of antioxidants during hot-air drying likely compromises oxidative stability, as previously reported by [22]. Importantly, freeze-drying maintains functional β-glucan matrices, which play a crucial role in physically impeding oxygen diffusion and lipid peroxidation. Recent research has demonstrated that these preserved structures are particularly effective at maintaining product stability during storage [23].

#### 2.1.4. Fatty Acid Composition

As an oily freshwater fish, carp meat has a rich and varied fatty acid profile, particularly in long-chain polyunsaturated fatty acids (LC-PUFAs), which are either absent or present in trace amounts in mushroom samples (Table 3). These include arachidonic acid (AA, C20:4 ω-6), docosahexaenoic acid (DHA, C22:6 ω-3), and eicosapentaenoic acid (EPA, C20:5 ω-3). Carp’s lipids profile reflects the typical lipid composition of freshwater fish, which is known for its contribution to the dietary intake of essential ω-3 fatty acids and the cardiovascular health benefits [24]. On the other hand, both hot-air and freeze-dried oyster mushrooms contain lower overall fat content, but their PUFA fraction constitutes a significant proportion of their total fatty acids. The primary polyunsaturated fatty acid (PUFA) found in mushrooms is linoleic acid (C18:2 ω-6), which is consistent with research findings indicating that mushroom cell membranes contain substantial amounts of linoleic acid [25]. Freeze-dried mushrooms contained significantly more linoleic acid (18.10%) than hot-air dried samples (15.05%). This difference reflects the lower water content in the case of freeze-drying and better preserves unsaturated lipid components due to the low-temperature and oxygen-limited process [26]. This effect is also reflected in the slightly higher total fatty acid content observed in freeze-dried mushrooms compared to hot-air dried ones (25.80 and 22.73 mg/g fat, respectively). Omega-3 PUFA were present in carp meat in a larger proportion compared to omega-6 PUFA by a ratio of 1.1:1, whereas neither of the mushroom samples has measurable amounts of long-chain ω-3 fatty acids. This shows how fish and mushrooms may function seamlessly in food formulations, with fish providing necessary ω-3 PUFAs and mushrooms potentially adding texture, bioactive substances, and unsaturated lipids.

### 2.2. Fish Burgers with Hot-Air Dried and Freeze-Dried Oyster Mushrooms

#### 2.2.1. Basic Characteristics

The addition of dried oyster mushrooms to fish burgers significantly influenced the changes in water, protein, lipid, and ash content (Table 4). Samples with the highest content of mushrooms (2%) have the highest water and ash content but the lowest protein and fat content. The reduction in protein is primarily a dilution effect, as part of the carp meat is replaced with oyster mushroom powder, which has a substantially lower protein concentration on a dry-weight basis. As the mushroom powders are rehydrated before incorporation, the additional water also increases the total mass of the mixture, further lowering the protein and fat value when expressed on a wet-weight basis. Conversely, the ash content increased proportionally to the level of mushroom inclusion. This effect reflects the naturally higher mineral content of oyster mushrooms compared with fish muscle [7]. Similar relationships are observed in our previous studies [4,27] and other scientific works on the use of oyster mushrooms in various food products [13,14]. Comparing hot-air drying and freeze-drying, the freeze-drying method of mushroom preservation contributes to a more significant enrichment of the finished product. Similar results are obtained by Das et al. (2020) [12], who also compared the effect of adding hot air-dried and freeze-dried oyster mushrooms on the properties of sponge cake.

#### 2.2.2. Determination of Total Glucans Content

The control sample showed a low amount of glucans—0.60 ± 0.05 g/100g DW, including 0.58 ± 0.06 g/100g DW of β-glucans (Table 5). Since no mushroom material is added to the control sample, this glucan content originates from the oat flour used as a binder. Oats are one of the richest cereal sources of mixed-linkage β-(1→3)(1→4)-D-glucans, and the literature consistently reports their β-glucan content at approximately 3–6% of dry matter, with values around 4% being typical for conventional oat flours used in food processing [28]. Research Majumdar et al. (2024) further emphasize that oat β-glucan content is strongly influenced by milling fraction, cultivar, and processing, but generally remains within this established range [29]. Therefore, even a modest addition of oat flour can contribute detectable background β-glucan levels in complex food matrices such as fish burgers. Freezing-drying is a safer technique for maintaining total glucans content in the samples when comparing the two techniques of processing oyster mushrooms. When comparing the two mushroom processing methods, freeze-dried material consistently yielded higher glucan levels in the fish burgers than hot-air dried material. This outcome is explained by the thermal sensitivity of mushrooms polysaccharides: hot-air drying exposes mushrooms to prolonged heating, which can degrade β-glucan chains and reduce measurable glucans content, whereas freeze-drying avoids these degradative conditions and therefore preserves glucans more effectively [30]. As a result, burgers containing freeze-dried mushrooms exhibited nearly double the glucan content of those produced with hot-air dried mushrooms at the same inclusion level. The highest glucans content (5.80 ± 0.08 g/100g DW) is found in the fish burger with 2% addition of freeze-dried oyster mushrooms, of which 96.90% are β-glucans. This significant proportion suggests that freeze-drying effectively preserves mushroom β-glucans [31] and allows for their efficient extraction from the burger matrix. Conversely, the reduced glucan levels observed in hot-air dried treatments indicate losses related to thermal processing.

#### 2.2.3. Determination of Lipid Quality Parameters

Traditional burgers, due to their significant fat content (approximately 20–30%), pose a challenge for consumers in the context of the growing awareness of the impact of diet on health [32]. The fat composition analysis in these products underscores the need to incorporate nutritional education into the decision-making process regarding food choices. In contrast to their meat counterparts, it is worth noting that fish burgers feature a lower fat content, around 10% of the product [4].

**Figure 1:**
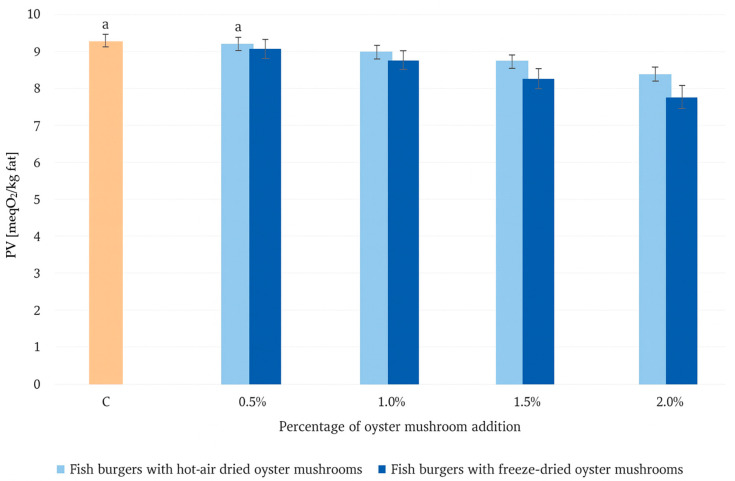
Comparison of peroxide values in fish burgers with hot-air dried and freeze-dried oyster mushrooms. Means in a bar with the same superscript letter do not differ significantly (*p* < 0.05). Lowercase letter indicate statistically significant differences. If no letter accompanies a value, this indicates that the value is significantly different from all other values within that row.

Figure 1 shows a comparison of the peroxides and hydroperoxides expressed as peroxide value—PV (meqO_2_/kg fat). The peroxide value in the control sample is 9.2 ± 0.0 meqO_2_/kg fat. In both cases, a statistically significant decrease in the peroxide value can be observed after adding 1% of hot-air dried or freeze-dried oyster mushrooms. When comparing drying methods, burgers containing freeze-dried oyster mushrooms consistently show lower PV than those containing hot-air dried samples. These reductions correspond to decreases of approximately 2.1%, 2.9%, 6.1%, and 9.7% relative to the control sample at inclusion levels of 0.5%, 1.0%, 1.5%, and 2.0%, respectively. The polysaccharides leached from the freeze-dried oyster mushroom matrix interact with fish myofibrillar proteins and β-glucans, forming a cohesive, dense network that effectively impedes oxygen diffusion [33]. This phenomenon is confirmed by the significantly lower peroxide values observed in freeze-dried compared to their hot-air dried samples.

**Figure 2:**
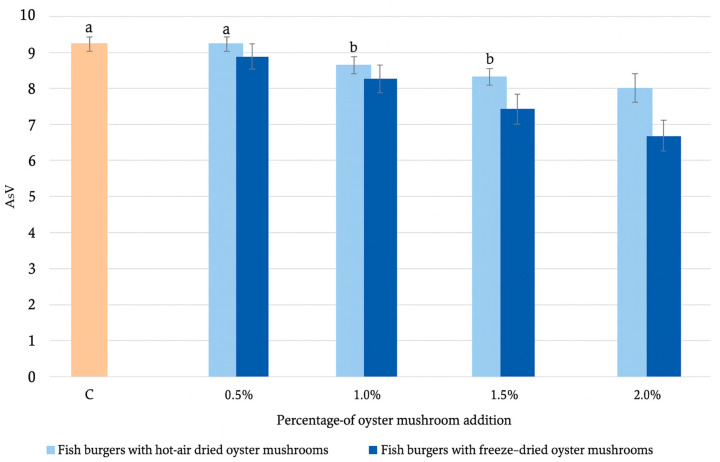
Comparison of anisidine values in fish burgers with hot-air dried and freeze-dried oyster mushrooms. Means in rows with the same superscript letter do not differ significantly (*p* < 0.05). Different letters indicate statistically significant differences. If no letter accompanies a value, this indicates that the value is significantly different from all other values within that row.

Figure 2 shows anisidine values (AsV), which reflect the concentration of secondary lipid oxidation products—primarily alk-2-enals and alkyl-2,4-dienals—formed during peroxide decomposition. According to the International Fish Oil Standard (IFOS) (n.d.), high-quality fish oil has an anisidine value of ≤20.00. The AsV for the control sample is 9.0 ± 0.1, meeting IFOS, and with the addition of hot-air dried or freeze-dried oyster mushrooms to the fish burger, this value decreases [34]. In the case of the control sample and a 0.5% addition of hot-air dried oyster mushroom, statistically significant changes are not observed (9.0 ± 0.0). This is attributed to the superior preservation of antioxidant compounds (E.G., Phenolic acids, peptides) in the freeze-dried mushroom powder [35], which effectively scavenge radicals and sequester reactive aldehydes, leading to the observed reduction in anisidine value. Compared with formulations containing hot-air dried mushrooms, the AsV in burgers with freeze-dried mushrooms is reduced by approximately 4.8%, 7.7%, 15.5%, and 22.8% at inclusion levels of 0.5%, 1.0%, 1.5%, and 2.0%, respectively. In the study by Hocine et al. (2022) [36] regarding the oxidative stability of beef, chicken, and shawarma burgers, the anisidine value ranged from 14.8 ± 0.2 for chicken shawarma to 48.5 ± 0.3 for chicken burgers. In our study, significantly lower results are achieved.

TOTOX values of lipids (Table 6) in the control sample (27.0 ± 0.0), the fish burger with a 0.5% addition of hot-air dried oyster mushroom (27.2 ± 0.1), and with freeze-dried oyster mushroom (26.4 ± 0.0) are higher than 26, according to IFOS (n.d.) standards [34]. However, with increasing addition of oyster mushroom, the anisidine value decreases, and in the burgers with 2.0% of mushrooms, it decreases to the level of 24.2 ± 0.1 for samples with hot-air dried oyster mushroom and 21.0 ± 0.0 for samples with freeze-dried oyster mushroom. This difference is due to the thermal degradation of antioxidants during hot-air drying [37], whereas freeze-drying retains β-glucans and phenolics that chelate pro-oxidant metals (E.G., Iron) and disrupt radical chain reactions [22].

**Figure 3:**
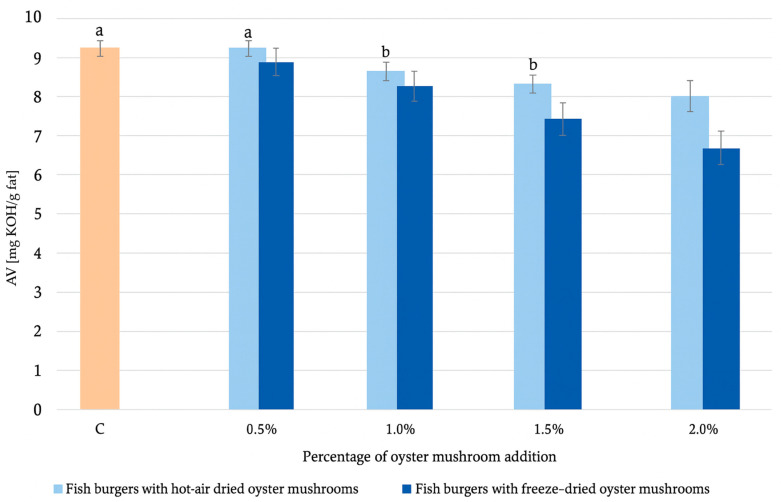
Comparison of hydrolytic changes in fish burgers with hot-air dried and freeze-dried oyster mushrooms. Means in a bar with the same superscript letter do not differ significantly (*p* < 0.05). Different letters indicate statistically significant differences. If no letter accompanies a value, this indicates that the value is significantly different from all other values within that row.

The degree of fat hydrolysis is measured by the amount of free fatty acids in 1 g of fat and is expressed as AV (mg KOH/g fat). The acid value is assessed to determine the quality of fats. The control sample is an AV of 5.3 ± 0.0 mg KOH/g fat, which is not significantly different statistically from all samples with the addition of hot-air dried oyster mushroom (Figure 3). A more significant reduction in AV is observed in samples with the addition of freeze-dried oyster mushrooms, which successively decreased by 0.4%, 4.3%, 8.9%, and 16.9%. Comparable AV were reported by Byun et al. (2008) [38] for fish oil extracted from yellowfin sole (4.8 ± 0.1 mg KOH/g), indicating that the reduction observed in freeze-dried mushroom-supplemented samples is within the range of high-quality fish oils. Although hydrolysis is mechanistically distinct from oxidation, previous studies have shown that bioactive compounds in plant and mushroom matrices can also limit hydrolytic lipid degradation, likely by reducing the availability of water, stabilizing the fat–protein network, or inhibiting lipolytic enzymes [39].

#### 2.2.4. Fatty Acids Composition

The fatty acid profile is presented in Table 7. In each sample, 28 fatty acids are identified. The total fatty acid content varied from 605.45 mg/g fat (for fish burgers with 2% hot-air dried oyster mushrooms) to 619.31 mg/g fat (for the control sample). The dominant fatty acids in all fish burgers are oleic acid (C18:1 ω-9), palmitic acid (C16:0), and linoleic acid (C18:2 ω-6). The addition of oyster mushrooms led to a general decrease in total lipid content, with a simultaneous decrease in SFA (saturated fatty acids) and variable increases in MUFA (monounsaturated fatty acids) and PUFA (polyunsaturated fatty acids). The reduction in SFA, particularly palmitic acid, and the relative increase in unsaturated fatty acids suggest a beneficial change in the nutritional quality of the lipid profile. These changes, while partially expected based on the mushroom composition and inclusion of oil in the preparation process, are further influenced by the drying method used for mushroom processing. According to Husein et al. (2019), in studies of the properties of fish burgers, including those made of Rainbow trout, the dominant acids among 12 identified are oleic and linoleic acids, with EPA content at 4.55% and DHA at 12.72% [40]. Interestingly, samples enhanced with hot-air dried mushrooms show a distinct increase in PUFA content, up to 21.25% with the addition of 1.5% hot-air dried oyster mushroom powder. In contrast, no similar trend is observed in freeze-dried samples, where PUFA content fluctuated between 20.47% and 21.36% across increasing concentrations. The differences observed between hot-air dried and freeze-dried mushroom treatments likely reflect the distinct effects of the two drying methods on the composition of the added raw materials. Hot-air drying exposes mushrooms to elevated temperatures that may modify lipid-associated components and influence the distribution of fatty acids in the final product [41]. In contrast, freeze-drying minimizes thermal effects and better preserves the native composition of the dried material. These differences in processing may contribute to the higher PUFA proportion noted in the samples containing hot-air dried mushrooms, whereas PUFA levels remained relatively stable across the freeze-dried treatments [42]. Hot-air drying partially degrades β-glucans, exposing hydrophobic sites that complex with and protect PUFAs [43], while generating Maillard products that stabilize ω-6 acids [44]. This explains both the PUFA increase and lower ω-3/ω-6 ratios (0.19–0.23) in hot-air dried burgers [45]. Although freeze-dried mushrooms improved the oxidative stability of the final products—as reflected by lower PV and AsV values compared with hot-air dried samples—the structural characteristics of the freeze-dried matrix interact differently during frying. Its higher porosity can facilitate oxygen diffusion under high-temperature conditions, potentially enhancing the susceptibility of ω-3 PUFAs to heat-induced oxidation [46]. Although no formal correlation analysis is performed, the experimental trends indicate an inverse relationship between β-glucan content and PUFA oxidative degradation, as samples with higher β-glucan levels consistently exhibited lower secondary oxidation markers.

Freeze-dried mushroom-enriched fish burgers exhibited a lower index of atherogenicity (AI) (0.18–0.36) and index of thrombogenicity (TI) (0.55–0.59) compared to hot-air dried samples, suggesting reduced cardiovascular risk. This is attributed to the preservation of MUFAs (e.g., C18:1 ω9) and PUFAs (e.g., C18:2 ω6, C20:5 ω3) in freeze-dried formulations, which counteract the atherogenic effects of SFAs [47]. The HH ratio reflects the balance between cholesterol-lowering (hypocholesterolemic) and cholesterol-raising (hypercholesterolemic) fatty acids. Freeze-dried samples demonstrated higher HH values (3.97–4.26 and 3.97–4.15 in hot-air dried), indicating a more favorable lipid profile for cardiovascular health. The improved lipid quality indices resulting from this preserved fatty acid profile are complemented by the presence of intact β-glucans from the freeze-dried oyster mushrooms. While β-glucans do not alter the fatty acid composition of the food itself, their well-documented role in increasing cholesterol excretion by binding bile acids [48] represents a separate, additive mechanism through which these fortified fish burgers may promote cardiovascular health after consumption. The Health-Promoting Index (HPI), which integrates multiple lipid quality parameters, is higher in freeze-dried formulations (2.71–3.20 and 2.71–3.14 in hot-air dried), enhancing their superior nutritional quality. This is consistent with previous studies showing that low-temperature processing better retains bioactive lipids that contribute to metabolic health [44]. The flesh lipid quality (FLQ) index decreased in fish burgers with added oyster mushrooms, especially when incorporating freeze-dried forms. The results range from 0.41 to 0.90 for the control sample. While β-glucans do not increase EPA or DHA content, their presence may contribute to an improved overall lipid profile [49].

## 3. Materials and Methods

### 3.1. Materials

The main ingredient for the burger production was mechanically separated meat from common carp. Carp (*Cyprinus carpio*, L.) were obtained in November from a local fish farm in the West Pomeranian Voivodeship, where they were harvested from Lake Ińsko (Ińsko, West Pomeranian Voivodeship, Poland). The fish were transported on ice to the laboratory in expanded polystyrene containers with a 25 kg capacity (Atlantic Styro A/S, Morawica, Świętokrzyskie Voivodeship, Poland). After being delivered to the laboratory, the fish were beheaded, gutted, and cooled with chilled potable tap water. The carcasses were then placed spine-up on perforated trays to drain. Subsequently, they were passed through a drum separator (NF 13DX Bibun, Fukuyama, Japan, with a 4.0 mm hole diameter) and then cleaned thoroughly in a screw separator (SUM 420, Bibun, Fukuyama, Japan, with a 2.5 mm hole diameter). That was how clean comminuted fish meat was received, which was used to produce fish burgers.

Oyster mushrooms (*Pleurotus ostreatus*) were obtained from G&G FUNGI (Salamony, Greater Poland Voivodeship, Poland) and processed immediately after delivery to the laboratory. First, the mushrooms were cleaned by gently brushing to remove visible substrate residues and surface impurities, followed by rinsing under cold running water and blotting dry with sterile paper towels. Next, mushrooms were cut into pieces approximately 0.5–1 cm wide. Some of the oyster mushrooms were hot-air dried using the natural convection dryer (Stalgast, Warsaw, Masovian Voivodeship, Poland) at 70˚C for 8 h, to achieve a water activity of approximately 0.300. The rest of the oyster mushrooms were first frozen to −20 °C and the next day freeze-dried at a temperature of −70 °C under reduced pressure of 0.027 mBar using the Bioevopeak Co., Ltd. FreezeDry LYO60B-1P (Jinan, Shandong, China) to obtain a humidity in the range of 1 to 2% by weight (approximately 36 h). Subsequently, the hot-dried and freeze-dried oyster mushrooms were ground to the state of powder using a laboratory high-speed multifunction grinder HC-350Y (Chemland, Stargard, West Pomeranian Voivodeship, Poland), vacuum packaged using a vacuum packaging machine ECOMAT (Webomatic, Bochum, Germany), and stored in a freezer at −20 ± 1 °C until analysis, which was used to produce fish burgers.

### 3.2. Preparation of Fish Burgers

Before preparing, the comminuted fish meat was stored in a refrigerator at 4 ± 1 °C.

Three completely independent batches of the burger mixture were made by combining comminuted carp meat (85.00% by weight) with oat flour (3.20%), rehydrated egg powder (0.80% in 2.40% of water), tomato concentrate (3.20%), various seasonings including dried garlic (0.15%), black pepper (0.07%), herb pepper (0.15%), hot pepper (0.02%), salt (1.00%), and rapeseed oil (4.00%). All ingredients were purchased at a local Intermarche store (Szczecin, West Pomeranian Voivodeship, Poland).

Before incorporation into the burgers, the dried oyster mushrooms were rehydrated at a constant rehydration coefficient (RF), corresponding to a 5:1 ratio of water to dried mushroom. Rehydration was performed at room temperature using tap water in accordance with methodologies applied in rehydration studies of dried plant materials [50]. After full water absorption, the samples were mixed into a homogeneous mass and immediately used to prepare burgers. The rehydration coefficient was calculated as the ratio of the mass of water added to the mass of dry raw material using the formula:

$$RF=\frac{Weight\;of\;water\;added}{Weight\;of\;dry\;sample}$$

From each batch, the control burgers (C) (without oyster mushrooms) and all mushroom-enriched variants were produced, with carp meat partially replaced by hot-air dried or freeze-dried oyster mushroom powder at inclusion levels of 0.5%, 1.0%, 1.5%, and 2.0%. This resulted in nine sample types, each prepared in triplicate as independent batches.

All these ingredients were homogenized thoroughly and shaped using the automatic burger press C/E H SMART (Resto Quality, Cracow, Lesser Poland Voivodeship, Poland). The resulting burgers, with dimensions of 7 cm in diameter, 10 mm in height, and a weight of 70 g, were then fried in a frypan containing hot rapeseed oil (170 ± 2 °C) until the internal temperature reached 75 ± 2 °C (which took approximately 5 ± 1 min). After draining excess oil, the burgers were cooled to room temperature (20 ± 1 °C) and then stored in a cold store at 4 ± 1 °C until the next day, when physicochemical analyses were performed.

### 3.3. Methods

#### 3.3.1. Proximate Analysis

The moisture content was determined by drying the sample in an oven at 105 °C until a constant weight was achieved [51]. The crude protein content was determined using the Kjeldahl method, and a conversion factor of 6.25 was applied to convert total nitrogen to crude protein [51]. Ash content was determined by ashing the samples in an oven at 550 °C for 8–12 h [51]. Results were expressed in g/100g of dry matter. The dry-matter basis was calculated from the recorded mass before and after the drying or ashing steps, where the final dry or ash mass served as the denominator. Lipid content was determined using the Bligh and Dyer method [52]. Single-phase lipid solubilization with a chloroform-methanol mixture (1:1) was used. Quantification results were expressed as grams of lipid per 100 g of product.

#### 3.3.2. Total Glucans Content

The content of total glucans, α-glucans, and β-glucans, was determined using the Assay Procedure for Mushroom and Yeast β-glucan K-YBGL 02/21 [53]. For total glucans, samples were first solubilized in ice-cold 12 M H_2_SO_4_. After complete dissolution, water was added to reduce the acid concentration to 2 M, in which the hydrolysis step was carried out. The partially hydrolyzed glucans were subsequently digested to glucose using purified exo-1,3-β-glucanase and β-glucosidase, and the released glucose was quantified using GOPOD reagent. Determination of α-glucans was performed separately by enzymatic hydrolysis with amyloglucosidase and invertase, which convert α-linked glucose polymers (including starch and glycogen) and sucrose into glucose and fructose. The released glucose was measured with GOPOD, providing the α-glucan value. β-Glucan content was calculated as the difference between total glucans and α-glucans. Results were expressed as g/100g dry matter.

#### 3.3.3. Determination of Lipid Quality Parameters

Peroxide value (PV) of the lipid extract [54] was determined using the method according to EN-ISO 3960:2017 [55] based on the iodometric determination of iodine liberated by the peroxides with a starch indicator and a sodium thiosulfate standard solution. Results were expressed as milliequivalents of active oxygen per kilogram of lipids (meqO_2_/kg of lipids).

Anisidine value (AsV) of the lipid extract [54] was determined according to the EN-ISO 6885:2016 method [56]. The method is based on the reaction between secondary lipid oxidation products, primarily alk-2-enals and alkyl-2,4-dienals, and *p*-anisidine reagent. AsV was expressed as 100 times the absorbance measured at 350 nm (Thermo Scientific, Waltham, MA, USA, Genesys 20) in a 1 cm path length cuvette from a solution containing 10 mg of lipid in 1 mL of reaction medium.

Total oxidation index (TOTOX) of the lipid phase of the products was calculated as follows:

2×PV+AsV

Acid value (AV) was determined by titration of 0.1 N KOH in methanol, according to EN ISO 660:2020 [57].

#### 3.3.4. Fatty Acids Composition (FAs)

Fatty acid methyl esters (FAMEs) were derived from the samples through alkaline hydrolysis of the lipid extract [52] using 0.5 N sodium methoxide (CH_3_ONa) [58], coupled with a mass spectrometer (Agilent Technologies 7890A, Santa Clara, CA, USA, Subsequently, the FAMEs were separated using a gas chromatography apparatus USA), and equipped with a split/splitless type injector. The separation conditions for FAME are as follows: SPTM 2560 column, with dimensions of 100 m length, 0.25 mm ID, and 0.20 μm film (catalog no. 24056); helium served as the carrier gas at a constant flow rate of 1.2 mL/min; the split ratio of 1:50; injector temperature set at 220 °C; detector temperature at 220 °C; programmed oven temperature starting at 140 °C (held for 5 min) and increasing to 240 °C at a rate of 4 °C/min; total analysis time of 45 min [55]. The qualitative interpretation of chromatograms was based on comparing retention times and mass spectra of specific FAMEs in the sample with those of analogous FAME standards by Sigma Company, Tokyo, Japan (Lipid Standard). The analyses were conducted in triplicate, and the tables presenting the results are the mean values. C19:0 (CAS 646-30-0, Merck, Warsaw, Poland) was the internal standard.

#### 3.3.5. Nutritional Quality Indices of Lipids

Seven indices were calculated using the fatty acid composition for each type of fish burger. The DHA/EPA index represents the ratio of docosahexaenoic acids to eicosapentaenoic acids. The PUFA/SFA index represents the polyunsaturated to saturated fatty acids ratio. The n-3/n-6 index represents the ratio of the sum of n-3 polyunsaturated fatty acids to the sum of n-6 polyunsaturated fatty acids [17]. The index of atherogenicity, index of thrombogenicity, hypocholesterolemic/hypercholesterolemic ratio, and fish lipid quality/flesh lipid quality were also determined and are described below.

- Index of Atherogenicity (AI)

The Index of Atherogeneity was calculated based on the formula developed by Ulbricht and Southgate (1991) [59].

$$AI=\frac{C12:0+\left(4\times C14:0\right)+C16:0}{\sum MUFA}$$

- Index of Thrombogenicity (TI)

The Index of Thrombogenicity was calculated based on the formula developed by Ulbricht and Southgate (1991) [59].

$$TI=\frac{C14:0+C16:0+C18:0}{\left(0.5\times \sum MUFA\right)+(0.5\times \sum n-6PUFA)+(3\times \sum n-3PUFA)+(\frac{n-3}{n-6})}$$

- Hypocholesterolemic/Hypercholesterolemic (HH) Ratio

The hypocholesterolemic/hypercholesterolemic ratio was calculated based on the formula of Zhang et al. 2020 [60]. Compared to the PUFA/SFA ratio, the HH ratio can more precisely reflect the impact of fatty acid composition on the cardiovascular system.

$$HH=\frac{C18:1n-9+C18:2n-6+C18:3n-3+C20:4n-6+C20:5n-3+C22:5n-3+C22:6n-3}{C14:0+C16:0}$$

- Fish Lipid Quality/Flesh Lipid Quality (FLQ)

Fish Lipid Quality/Flesh Lipid Quality was calculated based on the formula developed by Chen and Liu (2020) [17].

$$FLQ=\frac{100\times (C22:6n-3+C20:5n-3)}{\sum Total\;FA}$$

- Health-Promoting Index (HPI)

The Health-Promoting Index was calculated based on the formula developed by Zhang at al. (2020) [61]

$$HPI=\frac{\sum MUFA}{C12:0+4\times C14:0+C16:0}$$

#### 3.3.6. Statistical Analysis

All experiments and tests were conducted in triplicate, and the results were presented as the mean value ± the standard deviation (SD). Statistical analyses were performed using Statistica 13.1 software (StatSoft Polska, Cracow, Poland). A significance level of *p* < 0.05 is applied, and the analysis included a one-way analysis of variance, followed by Tukey’s Post Hoc test [62].

## 4. Conclusions

This study comprehensively evaluates the impact of different drying methods (hot-air drying and freeze-drying) on the nutritional quality and oxidative stability of oyster mushroom-enriched carp fish burgers. The results demonstrate that freeze-drying better preserves β-glucan content (up to 5.80 g/100g) and antioxidant capacity compared to hot-air drying, leading to significantly improved lipid stability as evidenced by 23% lower peroxide values and 35% reduced anisidine values in the final product. The incorporation of freeze-dried mushrooms at 1.5–2.0% concentration improved the nutritional profile by increasing the PUFA/SFA ratio by 15% while maintaining an atherogenicity index below 0.36. This research fills a significant gap in understanding the impact of processing techniques on the functional properties of mushroom ingredients in fish product applications, particularly regarding the dose-dependent effects on β-glucan retention and lipid protection mechanisms. These results provide practical guidance for developing functional fish products with enhanced nutritional value and longer shelf life, thus meeting the growing consumer demand for healthier protein alternatives. The study highlights the need for further investigation into the bioavailability of mushroom-derived bioactive compounds in processed seafood matrices to fully realize their health-promoting potential.

## Figures and Tables

**Table 1:** The basic compositions of carp meat [g/100g wet weight], hot-air dried and freeze-dried oyster mushrooms [g/100g dry weight].

	Carp Meat	Hot-Air Dried Oyster Mushrooms	Freeze-Dried Oyster Mushrooms
Water	75.2 ± 0.2	13.0 ± 0.8	0.6 ± 0.2
Protein	18.3 ± 0.3	22.5 ± 1.3 ^a^	24.6 ± 1.0 ^a^
Lipid	4.7 ± 0.4	0.8 ± 0.1 ^a^	1.0 ± 0.2 ^a^
Ash	1.0 ± 0.1	6.8 ± 0.2	7.8 ± 0.2

Means in rows with the same lowercase letter do not differ significantly (*p* < 0.05). Superscript letter indicate statistically significant differences. If no superscript letter accompanies a value, this indicates that the value is significantly different from all other values within that row.

**Table 2:** Values of lipid quality parameters in raw materials.

	Carp Meat	Hot-Air Dried Oyster Mushrooms	Freeze-Dried Oyster Mushrooms
PV [meqO_2_/kg fat]	5.1 ± 0.1	2.0 ± 0.0	1.1 ± 0.2
AsV	3.2 ± 0.0	0.5 ± 0.0	0.1 ± 0.0
AV [mg KOH/g fat]	0.6 ± 0.1	0.4 ± 0.0 ^a^	0.3 ± 0.1 ^a^
TOTOX	13.5 ± 0.3	3.5 ± 0.1	2.4 ± 0.2

PV—peroxide value; AsV—Anisidine value; AV—hydrolytic changes; TOTOX—total oxidation value. Means in rows with the same lowercase letter do not differ significantly (*p* < 0.05). Superscript letter indicate statistically significant differences. If no superscript letter accompanies a value, this indicates that the value is significantly different from all other values within that row.

**Table 3:** Fatty acid composition in raw materials [mg/g fat].

Fatty Acid	Carp Meat	Hot-Air Dried Oyster Mushrooms	Freeze-Dried Oyster Mushrooms
C10:0	0.10 ± 0.00	-	-
C12:0	0.20 ± 0.01	0.02 ± 0.00 ^a^	0.02 ± 0.01 ^a^
C14:0	0.04 ± 0.02 ^a^	0.11 ± 0.01	0.06 ± 0.00 ^a^
C15:0	0.03 ± 0.00	0.18 ± 0.02	0.10 ± 0.01
C16:0	75.45 ± 0.53	3.31 ± 0.20	1.96 ± 0.15
C16:1	13.74 ± 0.34	0.08 ± 0.02 ^a^	0.08 ± 0.01 ^a^
C17:0	2.14 ± 0.12	0.02 ± 0.01	0.06 ± 0.01
C17:1	0.89 ± 0.09	-	-
C18:0	219.96 ± 2.13	0.59 ± 0.11 ^a^	0.48 ± 0.10 ^a^
C18:1 ω-9	235.28 ± 1.64	3.25 ± 0.54	4.81 ± 0.48
C18:2 ω-6	15.76 ± 0.97 ^a^	15.05 ± 0.81 ^a^	18.10 ± 0.32
C20:0	0.46 ± 0.10	-	-
C18:3 ω-6	0.56 ± 0.09	-	-
C18:3 ω-3	2.24 ± 0.16	0.07 ± 0.02 ^a^	0.05 ± 0.01 ^a^
C20:1 ω-9	1.97 ± 0.04	0.06 ± 0.01 ^a^	0.07 ± 0.02 ^a^
C18:4 ω-3	1.01 ± 0.06	-	-
C20:2 ω-6	3.63 ± 0.06	-	-
C22:0	0.28 ± 0.01	-	-
C20:3 ω-6	0.42 ± 0.02	-	-
C22:1 ω-9	0.15 ± 0.01	-	-
C20:4 ω-6	0.76 ± 0.02	-	-
C20:4 ω-3	5.44 ± 0.21	-	-
C20:5 ω-3	1.21 ± 0.02	-	-
C22:4 ω-6	1.01 ± 0.01	-	-
C24:1 ω-9	1.12 ± 0.03	-	-
C22:5 ω-6	0.12 ± 0.01	-	-
C22:5 ω-3	2.32 ± 0.10	-	-
C22:6 ω-3	12.30 ± 0.12	-	-
SFA	298.66 ± 2.92	4.22 ± 0.34	2.69 ± 0.27
MUFA	253.04 ± 2.15	3.39 ± 0.55	4.96 ± 0.51
PUFA	46.89 ± 1.85	15.12 ± 0.85	18.15 ± 0.33
Total FA	598.59 ± 6.92	22.73 ± 1.74	25.80 ± 1.11
ω-3/ω-6	1.10	-	-

SFA—Saturated fatty acid; MUFA—Monounsaturated fatty acids; PUFA—Polyunsaturated fatty acids; FA—Fatty acids. Means in rows with the same lowercase letter do not differ significantly (*p* < 0.05). Superscript letter indicate statistically significant differences. If no superscript letter accompanies a value, this indicates that the value is significantly different from all other values within that row.

**Table 4:** The basic composition of fish burgers with hot-air dried and freeze-dried oyster mushrooms [g/100g wet weight].

	C	Fish Burgers with Hot Air-Dried Oyster Mushrooms				Fish Burgers with Freeze-Dried Oyster Mushrooms			
		0.5%	1.0%	1.5%	2.0%	0.5%	1.0%	1.5%	2.0%
Water	65.6 ± 0.1 ^a^	65.9 ± 0.2 ^a^	66.5 ± 0.1 ^a^	68.0 ± 0.2 ^b^	69.0 ± 0.1 ^b^	66.7 ± 0.2 ^a^	68.0 ± 0.1 ^b^	68.9 ± 0.1 ^b^	69.7 ± 0.1 ^b^
Protein	18.7 ± 0.2 ^c^	18.5 ± 0.1 ^c^	17.8 ± 0.1 ^b^	16.9 ± 0.1 ^a^	16.5 ± 0.1 ^a^	18.6 ± 0.1 ^c^	18.3 ± 0.1 ^c^	17.9 ± 0.1 ^b^	17.1 ± 0.9 ^a^
Lipid	9.7 ± 0.1 ^d^	9.6 ± 0.1 ^d^	9.3 ± 0.1 ^c^	8.9 ± 0.1 ^c^	8.4 ± 0.1 ^b^	9.2 ± 0.1 ^c^	8.3 ± 0.1 ^b^	8.0 ± 0.2 ^a^	7.8 ± 0.1 ^a^
Ash	2.8 ± 0.1 ^a^	2.9 ± 0.1 ^a^	3.3 ± 0.1 ^b^	3.6 ± 0.1	3.9 ± 0.1	2.9 ± 0.1 ^a^	3.1 ± 0.1 ^b^	3.2 ± 0.2 ^b^	3.2 ± 0.2 ^b^

Means in rows with the same lowercase letter do not differ significantly (*p* < 0.05). Different letters indicate statistically significant differences. If no superscript letter accompanies a value, this indicates that the value is significantly different from all other values within that row.

**Table 5:** Glucans (total glucans, α-glucans, and β-glucans) of fish burgers with hot-air dried and freeze-dried oyster mushrooms [g/100g dry weight].

	C	Fish Burgers with Hot-Air Dried Oyster Mushrooms				Fish Burgers with Freeze-Dried Oyster Mushrooms			
		0.5%	1.0%	1.5%	2.0%	0.5%	1.0%	1.5%	2.0%
Total Glucans	0.60 ± 0.05	1.79 ± 0.10	1.93 ± 0.13	2.06 ± 0.05	2.21 ± 0.06	4.24 ± 0.06	4.51 ± 0.04	5.10 ± 0.05	5.80 ± 0.08
α-Glucans	0.02 ± 0.03 ^a^	0.02 ± 0.02 ^a^	0.10 ± 0.06 ^b^	0.11 ± 0.09 ^b^	0.12 ± 0.08 ^b^	0.02 ± 0.10 ^a^	0.06 ± 0.10 ^ab^	0.09 ± 0.09 ^b^	0.18 ± 0.06
β-Glucans	0.58 ± 0.06	1.77 ± 0.11 ^a^	1.83 ± 0.11 ^a^	1.95 ± 0.06 ^b^	2.09 ± 0.08 ^b^	4.22 ± 0.08	4.45 ± 0.08	5.01 ± 0.06	5.62 ± 0.05

Means in rows with the same lowercase letter do not differ significantly (*p* < 0.05). Different letters indicate statistically significant differences. If no superscript letter accompanies a value, this indicates that the value is significantly different from all other values within that row.

**Table 6:** TOTOX values of lipids of fish burgers with hot-air dried and freeze-dried oyster mushrooms.

	C	Fish Burgers with Hot-Air Dried Oyster Mushrooms				Fish Burgers with Freeze-Dried Oyster Mushrooms			
		0.5%	1.0%	1.5%	2.0%	0.5%	1.0%	1.5%	2.0%
TOTOX	27.01 ± 0.03 ^b^	27.2 ± 0.1 ^b^	25.7 ± 0.2 ^a^	24.9 ± 0.0 ^a^	24.2 ± 0.1 ^a^	26.4 ± 0.0 ^b^	24.6 ± 0.2 ^a^	23.0 ± 0.0	21.0 ± 0.0

Means in rows with the same lowercase letter do not differ significantly (*p* < 0.05). Different letters indicate statistically significant differences. If no superscript letter accompanies a value, this indicates that the value is significantly different from all other values within that row.

**Table 7:** Fatty acid composition of fish burgers lipid fraction with hot-air dried and freeze-dried oyster mushrooms [mg/g fat].

	C	Fish Burgers with Hot-Air Dried Oyster Mushrooms				Fish Burgers with Freeze-Dried Oyster Mushrooms			
		0.5%	1.0%	1.5%	2.0%	0.5%	1.0%	1.5%	2.0%
C10:0	-	-	-	-	-	-	-	-	-
C12:0	0.03 ± 0.01 ^a^	0.04 ± 0.01 ^a^	0.02 ± 0.01 ^a^	0.05 ± 0.02 ^a^	0.05 ± 0.02 ^a^	0.04 ± 0.01 ^a^	0.03 ± 0.01 ^a^	0.04 ± 0.00 ^a^	0.03 ± 0.00 ^a^
C14:0	4.31 ± 0.02 ^c^	4.18 ± 0.04 ^c^	3.73 ± 0.04 ^b^	3.38 ± 0.01 ^b^	3.00 ± 0.03 ^a^	2.32 ± 0.02	4.31 ± 0.02 ^c^	4.69 ± 0.05	2.91 ± 0.05 ^a^
C15:0	0.39 ± 0.03 ^a^	0.44 ± 0.01 ^a^	0.36 ± 0.03 ^a^	0.31 ± 0.01 ^a^	0.28 ± 0.05 ^a^	0.22 ± 0.02 ^a^	0.39 ± 0.03 ^a^	0.46 ± 0.02 ^a^	0.32 ± 0.03 ^a^
C16:0	98.64 ± 0.04 ^b^	96.05 ± 0.01 ^a^	95.59 ± 0.02 ^a^	98.08 ± 0.00 ^b^	99.27 ± 0.02 ^b^	99.28 ± 0.03 ^b^	98.64 ± 0.04 ^b^	96.02 ± 0.03 ^a^	98.58 ± 0.02 ^b^
C16:1	10.44 ± 0.02 ^b^	11.57 ± 0.01 ^c^	9.35 ± 0.06	8.26 ± 0.02 ^a^	8.30 ± 0.02 ^a^	8.68 ± 0.02 ^a^	10.44 ± 0.02 ^b^	11.81 ± 0.02 ^c^	10.92 ± 0.03 ^b^
C17:0	1.23 ± 0.01 ^b^	1.34 ± 0.00 ^b^	1.12 ± 0.05 ^b^	0.96 ± 0.03 ^a^	0.82 ± 0.01 ^a^	0.58 ± 0.01	1.23 ± 0.01 ^b^	1.37 ± 0.02 ^b^	0.95 ± 0.04 ^a^
C17:1	1.23 ± 0.00 ^c^	1.08 ± 0.00 ^b^	0.90 ± 0.01 ^b^	0.58 ± 0.02 ^a^	0.65 ± 0.01 ^a^	0.49 ± 0.00 ^a^	1.23 ± 0.00 ^b^	1.11 ± 0.01 ^c^	0.85 ± 0.01 ^b^
C18:0	61.64 ± 0.01 ^b^	66.45 ± 0.02	59.71 ± 0.04 ^a^	55.60 ± 0.04	57.05 ± 0.09 ^a^	59.88 ± 0.00 ^c^	61.64 ± 0.01 ^b^	60.29 ± 0.09 ^a^	60.40 ± 0.01 ^a^
C18:1 ω9	279.76 ± 0.0 ^a^	278.75 ± 0.02 ^a^	295.00 ± 0.02 ^b^	293.46 ± 0.05 ^b^	295.90 ± 0.01 ^b^	295.52 ± 0.01 ^b^	299.76 ± 0.0 ^b^	296.31 ± 0.04 ^b^	298.84 ± 0.00 ^b^
C18:2 ω6	85.27 ± 0.06	97.12 ± 0.03	99.89 ± 0.03	94.10 ± 0.07 ^b^	95.10 ± 0.01 ^b^	94.42 ± 0.02 ^b^	95.27 ± 0.06 ^b^	90.66 ± 0.04 ^a^	90.30 ± 0.00 ^a^
C20:0	2.37 ± 0.05 ^b^	3.27 ± 0.04	2.22 ± 0.03 ^b^	1.89 ± 0.06 ^a^	1.75 ± 0.00 ^a^	1.40 ± 0.03	2.37 ± 0.05 ^b^	2.70 ± 0.02 ^c^	2.75 ± 0.00 ^c^
C18:3 ω6	0.76 ± 0.03 ^b^	0.43 ± 0.04 ^a^	0.85 ± 0.03 ^b^	0.93 ± 0.06 ^c^	1.00 ± 0.00 ^c^	0.96 ± 0.04 ^c^	0.76 ± 0.03 ^b^	0.32 ± 0.01 ^a^	0.38 ± 0.07 ^a^
C18:3 ω3	14.62 ± 0.03 ^a^	25.13 ± 0.05	18.15 ± 0.05 ^b^	19.90 ± 0.02 ^c^	19.67 ± 0.01 ^c^	17.68 ± 0.04 ^b^	14.62 ± 0.03 ^a^	20.51 ± 0.00 ^c^	18.57 ± 0.08 ^b^
C20:1 ω9	10.76 ± 0.02 ^a^	12.81 ± 0.01	10.51 ± 0.01 ^a^	9.86 ± 0.01	10.31 ± 0.03 ^a^	10.96 ± 0.05 ^a^	10.76 ± 0.02 ^a^	11.02 ± 0.00 ^b^	11.22 ± 0.06 ^b^
C18:4 ω3	0.27 ± 0.02 ^a^	0.41 ± 0.02 ^b^	0.30 ± 0.05 ^a^	0.29 ± 0.01 ^a^	0.28 ± 0.04 ^a^	0.25 ± 0.05 ^a^	0.27 ± 0.02 ^a^	0.37 ± 0.02 ^b^	0.31 ± 0.05 ^a^
C20:2 ω6	2.01 ± 0.01 ^a^	2.33 ± 0.01 ^b^	2.19 ± 0.00 ^a^	2.19 ± 0.00 ^a^	4.23 ± 0.02 ^c^	4.08 ± 0.02 ^c^	2.01 ± 0.01 ^a^	2.14 ± 0.04 ^a^	2.33 ± 0.05 ^b^
C22:0	0.71 ± 0.01 ^c^	1.21 ± 0.01 ^d^	0.52 ± 0.02 ^ab^	0.47 ± 0.03 ^a^	0.42 ± 0.03 ^a^	0.24 ± 0.01	0.71 ± 0.01 ^c^	0.53 ± 0.03 ^b^	0.63 ± 0.02 ^bc^
C20:3 ω6	1.36 ± 0.01 ^b^	1.62 ± 0.03 ^c^	1.36 ± 0.03 ^b^	1.33 ± 0.02 ^b^	1.13 ± 0.05 ^a^	2.24 ± 0.00 ^d^	1.36 ± 0.01 ^b^	1.50 ± 0.03 ^c^	1.23 ± 0.02 ^ab^
C22:1 ω9	0.59 ± 0.02 ^b^	1.51 ± 0.02	0.51 ± 0.04 ^ab^	0.48 ± 0.02 ^a^	0.45 ± 0.05 ^a^	0.43 ± 0.02 ^a^	0.59 ± 0.02 ^b^	1.26 ± 0.01 ^c^	1.20 ± 0.01 ^c^
C20:4 ω6	4.54 ± 0.02 ^c^	5.04 ± 0.04	4.41 ± 0.02 ^c^	3.20 ± 0.01 ^ab^	2.91 ± 0.01 ^a^	1.94 ± 0.02	4.54 ± 0.02 ^c^	4.49 ± 0.03 ^c^	3.50 ± 0.06 ^b^
C20:4 ω3	-	-	-	-	-	-	-	-	-
C20:5 ω3	0.68 ± 0.01 ^b^	0.74 ± 0.01	0.65 ± 0.07 ^b^	0.59 ± 0.01 ^b^	0.49 ± 0.04 ^ab^	0.41 ± 0.03 ^a^	0.68 ± 0.01 ^b^	0.69 ± 0.02 ^b^	0.53 ± 0.03 ^a^
C24:1 ω9	0.32 ± 0.03 ^b^	0.61 ± 0.02	0.38 ± 0.09 ^b^	0.32 ± 0.00 ^b^	0.27 ± 0.02 ^a^	0.19 ± 0.05 ^a^	0.32 ± 0.03 ^b^	0.30 ± 0.09 ^ab^	0.29 ± 0.03 ^a^
C22:4 ω6	0.43 ± 0.01 ^a^	0.68 ± 0.04	0.51 ± 0.05 ^a^	0.49 ± 0.07 ^a^	0.46 ± 0.02 ^a^	0.44 ± 0.01 ^a^	0.43 ± 0.01 ^a^	0.48 ± 0.03 ^a^	0.40 ± 0.03 ^a^
C22:5 ω6	0.63 ± 0.02 ^b^	0.76 ± 0.02	0.60 ± 0.02 ^ab^	0.53 ± 0.08 ^a^	0.42 ± 0.01	0.32 ± 0.02	0.63 ± 0.02 ^b^	0.68 ± 0.04 ^b^	0.59 ± 0.02 ^a^
C22:5 ω3	0.62 ± 0.03 ^b^	0.74 ± 0.02	0.61 ± 0.01 ^b^	0.57 ± 0.05 ^b^	0.53 ± 0.00 ^ab^	0.45 ± 0.03 ^a^	0.62 ± 0.03 ^b^	0.57 ± 0.05 ^b^	0.46 ± 0.01 ^a^
C22:6 ω3	4.58 ± 0.03 ^a^	4.81 ± 0.00	4.41 ± 0.00 ^a^	3.12 ± 0.02	2.81 ± 0.01	2.05 ± 0.02	4.58 ± 0.03 ^a^	4.22 ± 0.05	4.05 ± 0.00
SFA	28.81 ± 0.14	27.96 ± 0.40	26.61 ± 0.31 ^a^	26.96 ± 0.13 ^a^	26.78 ± 0.15 ^a^	27.08 ± 0.10 ^b^	27.38 ± 0.14 ^b^	27.07 ± 0.59 ^b^	27.11 ± 0.60 ^b^
MUFA	51.51 ± 0.65 ^a^	49.46 ± 0.51	51.58 ± 0.24 ^a^	51.69 ± 0.16 ^a^	51.97 ± 0.49 ^a^	52.24 ± 0.51 ^b^	52.27 ± 0.25 ^b^	52.46 ± 0.61 ^b^	52.61 ± 0.22 ^b^
PUFA	19.68 ± 0.26 ^a^	22.58 ± 0.15	21.82 ± 0.15 ^c^	21.35 ± 0.37 ^c^	21.25 ± 0.12 ^c^	20.68 ± 0.60 ^b^	20.35 ± 0.66 ^b^	20.47 ± 0.22 ^b^	20.23 ± 0.43 ^ab^
Total FA	588.37 ± 5.30	619.31 ± 5.84 ^b^	613.96 ± 3.28 ^b^	610.21 ± 3.82 ^a^	607.78 ± 3.04 ^a^	605.59 ± 4.92 ^a^	618.37 ± 5.10 ^b^	615.55 ± 4.93 ^b^	614.36 ± 5.51 ^b^
ω3/ω6	0.22	0.29	0.22	0.24	0.23	0.19	0.19	0.26	0.24
DHA/EPA	6.73	6.51	6.81	5.28	5.69	5.01	6.73	6.14	7.64
AI	0.38	0.18	0.35	0.36	0.35	0.34	0.36	0.36	0.34
TI	0.63	0.55	0.56	0.56	0.56	0.59	0.60	0.55	0.57
PUFA/SFA	0.68	0.81	0.82	0.79	0.79	0.76	0.74	0.76	0.75
HPI	2.61	2.71	2.86	3.08	3.09	3.11	3.14	3.20	3.18
HH	3.79	4.11	4.26	4.09	4.08	4.06	4.08	4.15	4.10
FLQ	0.89	0.90	0.82	0.61	0.54	0.41	0.85	0.80	0.75

SFA—Saturated fatty acid; MUFA—Monounsaturated fatty acids; PUFA—Polyunsaturated fatty acids; FA—Fatty acids; DHA—Docosahexaenoic acid; EPA—Eicosapentaenoic acid; AI—Index of atherogenicity; TI—Index of thrombogenicity; HPI—Health-Promoting Index; HH—Hypocholesterolemic/hypercholesterolemic ratio; FLQ—flesh lipid quality. Means in rows with the same lowercase letter do not differ significantly (*p* < 0.05). Different letters indicate statistically significant differences. If no superscript letter accompanies a value, this indicates that the value is significantly different from all other values within that row.

## Data Availability

No new data were created or analyzed in this study. Data sharing is not applicable to this article.
